# Fertility, gonadal and sexual function in survivors of nasopharyngeal carcinoma patients

**DOI:** 10.1186/s12885-025-14838-x

**Published:** 2025-10-15

**Authors:** Xiao Liu, Xuefeng Luo, Yang Xian, Lijuan Ying, Xiaofang Zhu, Yuanyuan Zeng, Siyu Long, Bo Liu, Fuping Li

**Affiliations:** Department of Andrology/Sichuan Human Sperm Bank, West China Second University Hospital, Sichuan University, Chengdu, China; Key Laboratory of Birth Defects and Related Diseases of Women and Children (Sichuan University), Ministry of Education, Chengdu, China

**Keywords:** Nasopharyngeal carcinoma, ﻿Male fertility, Semen parameter, Sexual dysfunction, Fertility

## Abstract

**Purpose:**

Therapy with radiotherapy in the head and neck, can be associated with gonadal damage in male survivors of cancer. To the authors’s knowledge the effect of treatments on testicular reproductive and endocrine function in nasopharyngeal carcinoma (NPC) patients has not been established.

**Methods:**

A retrospective study of NPC analyzed hormone levels, semen parameters, sexual functioning, fertility outcome before treatment and treatment after 0, 3, 6, and 12 months.

**Results:**

The incidence of NPC is high in Sichuan (9.5/100,000). Pre-treatment, 79.4% of NPC patients were normozoospermic. NPC were associated with worse total sperm number compared to healthy controls. There was no significant difference on sperm concentration between differentiated keratinizing group and undifferentiated non-keratinizing group. Post-treatment analyses showed that first-line treatments worsened at 0, 3 and 6 months after the end of treatment (T0, T3, T6), with total sperm number returning to previous level at 12 months.

Sexual functions were not significantly impacted by treatment modalities, except for poorer the problem assessment of drive, erection, and ejaculation problems (DEE problems). Fertility data were available for 44 patients: Seven patients (15.9%﻿) desired children after treatment. Six patients achieved fatherhood: 4 through natural conception and 2 following artificial reproductive techniques (ART).

**Conclusions:**

To the authors’ knowledge, this is the first time reported the study on fertility preservation in patients with NPC. Following the azoospermia/ oligospermia induced by personalized chemotherapy regimen with NPC, spermatogenesis May take 1-2 years to recover. Nasopharyngeal cancer treatment can affect erectile dysfunction. Awareness of this issue will enable oncologists to better inform patients about the possibility of recovering fertility post-treatment and also demonstrates the importance of semen cryobanking before beginning cancer treatment.

**Supplementary Information:**

The online version contains supplementary material available at 10.1186/s12885-025-14838-x.

## Introduction

Nasopharyngeal carcinoma (NPC) is a rare malignancy originating from nasopharyngeal mucosal lining [1–3]. According to the World Health Organization, there are three pathological subtypes of nasopharyngeal carcinoma: keratinizing squamous, non-keratinizing, and basaloid squamous [1]. The keratinizing Subtype is relatively rare in southern China and the non-keratinizing Subtype constitutes most cases in China, accounting for about 95.0% of all cases. Non-keratinizing nasopharyngeal carcinoma can be classified into differentiated and undifferentiated tumors [4]. According to Cancer Today (https://gco.iarc.who.int/today), in 2022, a total of 120,434 new cases from NPC were reported in world, with 17,238 adolescent and young adult (age 15 to 39) new cases [5]. The geographical global distribution of NPC is extremely unbalanced, mainly in Northern Africa and Southeast Asia, with a high incidence in China. The incidence of NPC is higher in males than in females, with a ratio of nearly three to five in China [6, 7]. According to Cancer Today, in 2022, there were about 510,10 new cases of nasopharyngeal carcinoma in China, with 5849 adolescent and young adult (age 15 to 39) new cases (about 33.9% of new cases are in China). Although the 5-year relative Survival rate of NPC in China is only 43.8% in 2003–2005 [8], with the improvement of medical technology, the Survival rate has increased to 75% in 2020 [9]. Survivors of NPC patients had an increasing demand for fertility and oncologists have begun to focus on fertility in NPC patients.

Cancer treatment-induced male gonadal damage has been studied in populations of hematologic tumor and testicular tumor survivors [10–16]. The main treatment of NPC is head and neck radiotherapy combined with systemic chemotherapy [17]. Cranial radiotherapy can damage effect on the hypothalamic–pituitary axis. For Males, even craniocerebral radiation dose reached 25 Gy can affect fertility [18, 19]. Systemic chemotherapy, which includes paclitaxel plus cisplatin, is the main chemotherapy regimen for nasopharyngeal carcinoma patients [20]. Pacilitaxel and cisplatin usually cause only temporary sterility [21–23], but they can also have an additive effect when combined with head and neck radiotherapy.

Few studies focus on research fertility in survivors of NPC in their reproductive years.

A previous study showed that pregnancy after NPC treatment was not associated with adverse clinical outcomes [24], but another research Suggested that among the female NPC Survivors, 11 of 16 (62.5%) had menstrual or fertility problems [25]. Furthermore, among males treated Nasopharyngeal radium irradiation more exposed than nonexposed men reported a history of fertility problems [26, 27]. To the best of our knowledge, there have been no detailed reports on the fertility (including semen quality, sex hormone levels, and sexual function) of male nasopharyngeal carcinoma survivors. So, it is important to assess the risk of infertility after NPC exposure to radiotherapy and chemotherapy. To furtherly investigate the effect of treatment on male gonadal function in NPC patients, we examined semen parameters, sexual function, fertility and hormone levels in a cohort of NPC male survivors. Clinicians can use this information to better counsel patients and evaluate of the NPC survivor’s reproductive function.

## Materials and methods

### Patients

The experimental protocol was established, according to the ethical guidelines of the Helsinki Declaration and was approved by Ethics Committee of West China Second University Hospital, Sichuan University (No: 2023 − 297). Written informed consent was obtained from individual or guardian participants.

The healthy controls in this study were healthy men who were candidates for sperm donation.

We performed a retrospective study by collecting 64 records of NPC patients who attempted sperm banking in the Human Sperm Bank, West China Second University Hospital, Sichuan University.

All patients and controls provided one semen sample (before treatment), and the patients were also asked to produce four other samples 0 months (T0, immediately after treatment ends), 3months (T3),6 months (T6), and 12 months (T12) after the end of treatment according to the study design (Supplemental Fig. 1).

The cryopreservation process for patients follows the regulations of the Sichuan Human Sperm Bank [28]. In short, before signing a written consent for cryopreservation, patients were fully informed regarding the procedure, including the process entailed, costs, future use, and storage duration. We followed up cancer patients by we-chat. The healthy controls in this study were men screened as potential sperm donors.

### Semen analysis

Semen samples (including healthy controls and NPC) were collected by Masturbation after 2–7 days of sexual abstinence. While a few of the patients had a longer period of sexual abstinence than one week, due to emergency treatment. All samples were allowed to Liquefy at 37℃ for 60 min and were then assessed in accordance with World Health Organization guidelines (WHO).

### Measurement of reproductive hormones

Blood samples were drawn to determine estrogen (E2), luteinizing hormone (LH), follicle-stimulating hormone (FSH), and testosterone (T) levels. The blood samples are sent to the Clinical laboratory of our hospital for testing.

### Sexual function

The Brief Male Sexual Function Inventory (BSFI) is a validated self-report measure of sexual functioning, first utilized in 1995 by O’Leary et al. [29]. This questionnaire consisted of 11 questions on sexual drive and satisfaction, erectile function, ejaculatory function, DEE problem, as well as overall sexual satisfaction. The item scaling is from 0 (no function, big problem, etc.) to 4 (good function, no problem, etc.). All responses in each section were totaled for a summary score [29]. Lower scores mean poorer function. Previous work suggested valid and reliable data from the BSFI [30–32].

### Statistical analysis

All relevant data and information were collected and a normality test was first performed on the measured data. Baseline data were presented using descriptive statistics (mean and SD for normally distributed variables or median and interquartile range for skewed variables). Continuous variables presented normal distribution, comparisons between two groups were performed using Student’s t-test. Continuous variables presented non-normal distribution, comparisons between two groups were performed using the Mann–Whitney U test. Semen parameters from follow-up of these patients were compared versus baseline using Friedman’s test. Hormone parameters from follow-up of these patients were compared versus baseline using ANOVA test.

Statistical analysis was performed using GraphPad Prism 5 (GraphPad Software). In all cases, statistical significance was set at *p* < 0.05.

## Results

### Population studied

The analyses included 64 nasopharyngeal carcinoma patients (mean age at diagnosis/cryopreservation 27.2 ± 7.4years), never married (74.2%), without children (85.0%), Bachelor’s degree/post-graduate’s degree (71.9%). Information is available in Table 1.

**Table 1:** Demographics of patients with nasopharyngeal carcinoma

Nasopharyngeal carcinoma study group			
Age at diagnosis (years)			27.2﻿±7.4
BMI (kg/m^2^)			23.4±5.4
﻿Marital status	Single, never married		74.2%
	Divorced or widowed		3.2%
	Married		22.6%
Bearing status	﻿Without children		85.0%
	﻿With children		15.0%
﻿Education	Bachelor's/Post-graduate degree		71.9%
	no bachelor's degree		28.1%

### Crude incidence rate of NPC in some china region

The incidence of NPC in China is higher than the global average level, showing a gradually increasing trend from north to south. The high incidence of NPC in China is concentrated in Guangzhou, Guangxi, Fujian, Jiangxi and Hunan. The incidence of NPC is obviously related to race. According to the relevant cultural and historical data of Sichuan Province, the immigrants Mainly come from Hubei, Hunan, Guangdong, Fujian and Jiangxi provinces, so the incidence of NPC is also high in Sichuan. The permanent resident population of Sichuan Province is 83.8 million. The cancer incidence rate for men in Sichuan Province is 289.7 per 100,000 in 2022 and the incidence of NPC is 9.5 per 100,000 (The data is from Tumor Registration Annual Report of Sichuan Province in 2022), comparing to Guangzhou (18.1), Guangxi (10.7), Hunan (7.1), Jiangxi (5.8) and Fujian (5.1) (Table 2).The percentage of males receiving sperm cryopreservation for NPC cancers (15–39 years old) in China in 2020 was only 0.4% [33]. The practice of fertility preservation is far from widespread among NPC patients.

**Table 2:** Crude incidence rate of nasopharyngeal carcinoma in some cancer registries (1/100,000)

Region	﻿Crude incidence rate	
	﻿Both sexes	Male
Guangdong	18.1	25.9
Guangxi	10.7	/
Hunan	7.1	9.6
Jiangxi	5.8	7.4
Fujian	5.1	7.2
Sichuan	**9.5**	**/**
National level	3.1	4.3

Data are values per 100 000 person-years

This table is come from annual report on tumor registration in each province

### Before NPC treatment (63 NPC patients and 100 controls)

One of the sixty-four selected NPC patients could not collect the semen sample for cryopreservation. 63 NPC patients have sperm and Success to cryostore. According to the WHO 2021 reference sixth percentile (total sperm number ≥ 39.0 × 10^6^/ejaculate), there are 58 (79.4%) normozoospermic patients. Sperm output for NPC patients was lower than healthy controls of similar age (Table 3). There are three pathological subtypes of NPC: keratinizing squamous, non-keratinizing, and basaloid squamous. The most frequent histological diagnosis was differentiated non-keratinizing nasopharyngeal carcinoma (52.4% of subjects), followed by unspecified (could not be definitively diagnosed, 38.1%), undifferentiated tumors (9.5%). No significant correlation was found between specific histological subtype and sperm concentration/reproductive hormones parameters (Table 4).

**Table 3:** Semen analysis results from Healthy controls and Nasopharyngeal carcinoma patients

	﻿Healthy controls	﻿Nasopharyngeal carcinoma	Reference value	*p-Value*
N	100	63		
Volume (mL)	**3.7**	**3.4**	1.4	0.0340
	(3.0-4.7)	(2.5-4.3)	(1.3-1.5)	
Count (10^6^/mL)	**131.5**	**72.0**	16.0	<0.0001
	(93.0-182.0)	(46.8-132.0)	(15.0-18.0)	
Total sperm number (10^6^)	**481.7**	**272.2**	39.0	<0.0001
	(367.1-662.9)	(119.6-406.0)	(35.0-40.0)	
Progressive motility (%)	**69.0**	**51.0**	30.0	<0.0001
	(65.0-76.0)	(42.3-64.0)	(29.0-31.0)	

Data are medians (in brackets) and 25th to 75th percentile distribution

Reference value: fifth percentile, fifth percentile given with variability (95% confidence interval)

The healthy controls in this study were men screened as potential sperm donors

**Table 4:** Semen analysis and hormone level results in men with Differentiated and Poor differential non-keratinizing nasopharyngeal

	Differentiated non-keratinizing nasopharyngeal carcinoma	Undifferentiated non-keratinizing nasopharyngeal carcinoma	Reference value	*p-Value*^c^
N	33	6		
Abstinence time（days)	5.0	7.0	NA	ns
	(4.3-7.0)	(6.3-7.8)		
Volume at banking (mL)	3.5	4.0	1.4	ns
	(2.6-4.4)	(3.5-4.6)	(1.3-1.5)	
Pre-count (10^6^/mL)	57.0	55.7	16.0	ns
	(39.0-138.5)	(5.6-130.7)	(15.0-18.0)	
Pre-progressive motility (%)	57.0	43.0	30.0	ns
	(44.0-65.0)	(34.5-51.5)	(29.0-31.0)	
Serum E2 (pg/ml)	25.5±10.3	29.9±12.9	0.0-39.8	ns
Serum T (ng/ml)	3.4±1.1	4.0±1.5	1.2-8.1	ns
Serum LH (IU/L)	4.5±2.9	4.5±2.2	1.5-9.3	ns
Serum FSH (IU/L)	4.5±2.2	4.8±1.6	1.4-18.1	ns

Data shown as mean and standard deviation

Reference value: fifth percentile, fifth percentile given with variability (95% confidence interval)

Pre: sperm before banking

### After NPC treatment (20 patients)

Treatment details were available for 20 patients. All patients had undergone field radiotherapy, different from chemotherapy regimen. Of these, 9 had undergone “TP + Cetuximab + Radiation” treatment, chemotherapy regimen (TP: pacilitaxel-cisplatin, Cetuximab) and field radiotherapy. 7 patients underwent treatments followed by “TP + Radiation” and 4 patients underwent “GP/DPF + Radiation” treatment chemotherapy regimen (GP: Gemcitabine-cisplatin, DPF: Docetaxel-cisplatin-5-Fu).

Total sperm number was worst significantly at 0 months after the end of treatment (T0), which in “TP + Cetuximab + Radiation” and “GP/DPF + Radiation” groups, all patients presented Azoospermia. Moreover, total sperm number worsened at 3 months after the end of treatment (T3), 6 months after the end of treatment (T6) compared to baseline (Tb). Twelve months after the end of treatment (T12), total sperm number did not significantly differ from pretreatment values, and they returned to normal values in all three groups (Table 5).

**Table 5:** Decrease in total sperm number from Tb to T12 against treatment regimen

Group	Patients (n)	Total sperm number (10^6^/ejaculate)										
		**Tb**	**T0**		**T3**		**T6**		**T12**			
TP+Cetuximab+Radiation	9	**60.0**	**0.0**		**1.2**^a^		**7.3**^a^		**45.0**			
		(50.8-98.0)	(0.0-0.0)		(0.6-4.3)		(4.1-20.5)		(4.02-101.3)			
TP+Radiation	7	**59.0**	**0.4**^a^		**2.0**^a^		**25.5**^b^		**85.0**			
		(54.5-253.5)	(0.1-0.7)		(1.6-25)		(10.0-49.0)		(67.0-102.5)			
GP/ DPF+Radiation	4	**61.0**	**0.0**		**3.1**^b^		**24.0**^b^		**57.0**			
		(61-65.5)	(0.0-0.0)		(2.1-35.0)		(12.0-35.0)		(26.0-87.5)			

Data are medians (in brackets) and 25th to 75th percentile distribution

a: *P* < 0.05 versus Tb

b: *P* < 0.01 versus Tb

Tb: before-treatment, T0, 3,6,12: after-treatment (0, 3, 6 and 12 months)

TP: Pacilitaxel-cisplatin

GP: Gemcitabine-cisplatin

DPF: Docetaxel-cisplatin-5-Fu

Our study found no difference in E2 and T level at T0, T3, T6, T12 compared at Tb.

After treatment, serum levels showed higher LH (non-significantly) and higher FSH (significantly) levels. Serum levels of FSH was increased at T0(*P* = 0.003), T3(*P* < 0.001), T6(*P* = 0.005) compared at Tb as shown in Table 6. Our findings are generally consistent with those of Kirsi Jahnukainen et al. suggesting testicular size and FSH were shown to be better in predicting fertility [34].

**Table 6:** Hormone level result from Tb to T12 against treatment regimen

	Tb	T0	T3	T6	T12	reference ranges
Serum E2 (pg/ml)	30.1±15.7	35.3±16.6	33.8±18.7	35.4±17.8	29.4±14.7	0.0-39.8
Serum T (ng/ml)	3.5±1.0	5.7±1.9	6.2±2.4	5.5±2.0	4.4±1.8	1.2-8.1
Serum LH (IU/L)	3.1±1.2	5.9±2.4	5.8±2.5	5.8±2.6	4.9±2.3	1.5-9.3
Serum FSH	5.3±2.8	16.1±9.2^a^	19.2±	13.2±	10.0±8.4	1.4-18.1
(IU/L)			13.1^a^	8.7^a^		

Data shown as mean and standard deviation

a: *P* < 0.01 versus Tb

Tb: before-treatment, T0,3,6,12: after-treatment (0, 3, 6, and 12 months)

### Sexual functioning

The BFSI is a validated self-report measure of sexual functioning. We compared BSFI scores before cancer treatment and after cancer treatment, no differences were observed in sexual drive, sexual erection, sexual ejaculation, and sexual satisfaction. However, after cancer treatment group significantly poorer than before cancer treatment group on DEE problems(*P* = 0.042). Sexual functions were not significantly impacted by treatment modalities, except for poorer DEE problems (Table 7).

**Table 7:** Demographic data and BSFI findings of the Nasopharyngeal carcinoma survivors

Demographics	Untreatment	Chemotherapy/Radiation	*P-value*
	(n=27)	(n=17)	
Age, mean (SD)	28.4 (6.8)	30.6 (8.8)	0.1760
BSFI, mean (SD)			
Drive	3.7 (1.3)	3.5 (1.7)	0.8703
Erection	8.1 (2.2)	7.0 (2.2)	0.1006
Ejaculation	7.4 (1.6)	7.1 (1.7)	0.1863
DEE problems	10.5 (2.7)	8.7 (3.6)	0.0420*
Satisfaction	3.7 (1.1)	3.6 (1.1)	0.7012

lower scores mean poorer function

Range of BSFI scores: drive = 0–8; erection = 0–12; ejaculation = 0–8

DEE problems = 0–12; satisfaction = 0–4

*SD* Standard deviation, *BSFI* Brief Male Sexual Function Inventory, *DEE* Drive, erection, ejaculation

### Fertility outcome

**Fig. 1:**
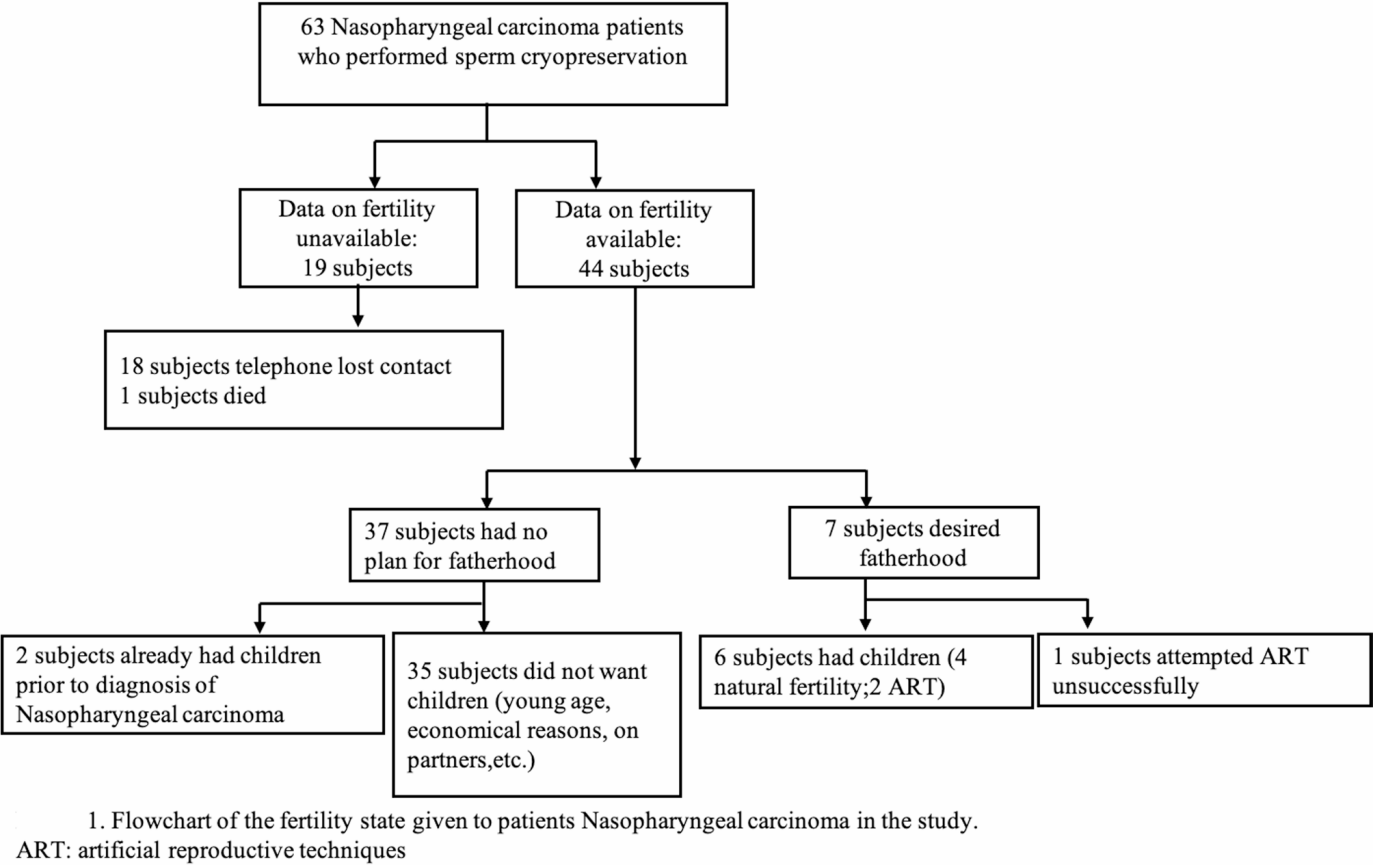
Flowchart of the fertility state given to patients nasopharyngeal carcinoma in the study. ART: artificial reproductive techniques

Fertility data were available for 44 patients. 19 patients were not included in this Survey: 18 did not answer the questionnaire and 1 died due to cancer progression. 35 (79.5%) had no children, for various reasons (young age, financial reasons, marital status), 2 (4.5%) already had at least one child prior to their cancer diagnosis. Seven patients (15.9%) wanted children, 6 of these succeeded in achieving fatherhood, through natural fertility (4 patients) or ART (2 patients) (Figure. 1).

## Discussion

There have been few published reports regarding fertility problems after chemoradiotherapy for NPC. Liqin Ma et al. reported that post-treatment birth did not increase the mortality risk of child-bearing women with NPC [35]. Suying Lu et al. showed that among all 14 male survivors, only one suffered from infertility [25]. We did not find any published study that specifically reviewed issues of semen parameter, reproductive hormones, sexual functioning, and fertility outcomes in men who had chemoradiotherapy for NPC. The present study demonstrates that NPC patients have not altered sperm parameters before cancer treatment compared with a control group of healthy men and that there was no significant correlation between specific histological subtype and sperm concentration/reproductive hormones parameters.

Cisplatin induced testicular damage such as germ cell apoptosis, steroidogenic impairment, and histological changes that resulted in damaging reproductive system [5, 6]. It stated that male patients receiving cisplatin dosages of 600 mg/m^2^ or greater will impaired spermatogenesis at medium risk [22]. Although NPC treated with dose cisplatin as part of a TP regimen no providing dose, our data showed that at the end of treatment of 0, 3, 6 months, total sperm number decreased significantly, suggesting the 1-year time threshold better to reproductive. Radiotherapy or surgery on the hypothalamic–pituitary–gonadal axis could result in adverse effects of antineoplastic therapies on the reproductive organs. A total dose of 24 Gy has relatively high risk of hypothalamic/pituitary dysfunction [36]. Although high-precision modern radiation therapy techniques (e.g., intensity modulated radiation therapy, IMRT) reduced the radiation in the surrounding area, the study about radiotherapy effecting on the reproductive of NPC patients was few. Our data showed that the reduction in total sperm number might be attributable to radiotherapy and chemotherapy. It showed that serum FSH combined with testicular size may offer a practical approach for predicting testicular damage for the treatment of malignancies during childhood [37]. If serum FSH is elevated then impaired sperm production is highly likely. Our findings also suggest that elevated serum FSH consistently was associated with an abnormal total sperm number.

To the best of our knowledge, few studies focus on sexual functioning in survivors of NPC. In this study, it reported treatment modalities did not influence sexual functions significantly, except for DEE problems, which was significantly poorer in treatment compared with un-treatment. Physical inactivity and chronic fatigue or anxiety disorder were moderately relevant for most sexual functions [31, 38].

Although the incidence of NPC is low in the world, the incidence is relatively high in southern China. Due to the early population migration, Sichuan is also a region with a high incidence in China, so this study will provide clinical practitioners with evidence-based insights for fertility preservation in nasopharyngeal carcinoma patients. We call on clinicians to quantify the reproductive toxicity of different treatment regimens while integrating patient age and fertility needs to assess gonadal toxicity risks. Fertility preservation may induce anxiety (e.g., concerns about technical efficacy), necessitating professional psychological support for patients. Additionally, long-term monitoring is required for pregnancy timing (recommended ≥ 1 year post-chemotherapy), pregnancy complications (e.g., elevated preterm birth rates), and tumor recurrence risks. The ASCO guidelines (2025) emphasize that all post-adolescent oncology patients should receive fertility counseling before treatment [39]. Patient consultations must balance scientific rigor, ethical considerations, and compassionate care. Future efforts should prioritize policy support, technological advancements, and public education to achieve the dual goals of ‘survival’ and ‘fertility’ for patients.

There are some Limitations in our study. We did not specifically collect the detailed pathological classification of NPC, so 38.1% NPC could not be provided definitively pathological classification. The other limitation is the small size of the cohort in this study. In comparison with other cancers, NPC is low incidence, and data on pregnancies are even more difficult to obtain because of the smaller number of people involved and the long-term follow up needed.

## Conclusions

In conclusion, our data indicate that comparison of pre- and post-therapy semen parameters and reproductive hormones, chemoradiotherapy for NPC is detrimental to spermatogenesis at T0, T3, T6, with recovery at T12. Young male patients should be better informed about the possibility of recovering fertility and semen cryobanking prior to therapy.

## Supplementary Information

Supplementary Material 1.

## Data Availability

All data generated or analyzed during this study are included in this published article.

## Abbreviations

NPC: Nasopharyngeal carcinoma patients
E2: Estrogen
LH: Luteinizing hormone
FSH: Follicle-stimulating hormone
T: Testosterone
BSFI: Brief Male Sexual Function Inventory
DEE problems: The problem assessment of drive, erection, and ejaculation
WHO: World Health Orgnization
TP: Pacilitaxel-cisplatin
GP: Gemcitabine-cisplatin
DPF: Docetaxel-cisplatin-5-Fu
ART: Artificial reproductive techniques
