# Supplementary figures and images for: Fertility, gonadal and sexual function in survivors of nasopharyngeal carcinoma patients
Source: BMC Cancer. 2025 Oct 15;25:1583. doi: 10.1186/s12885-025-14838-x (PMC12522230; doi:10.1186/s12885-025-14838-x)

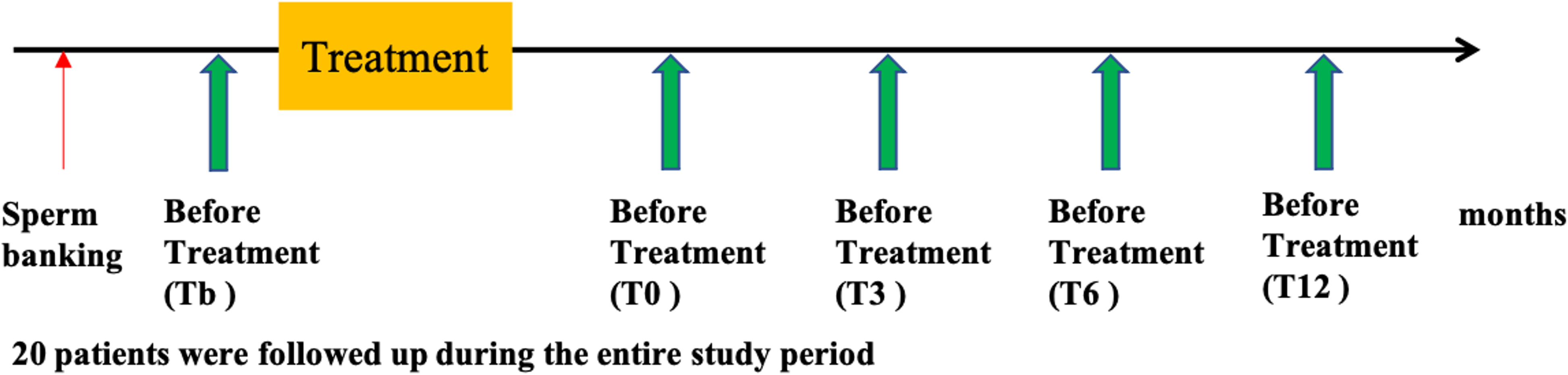

Supplement: Supplementary file 1 — Supplementary Material 1. [file 12885_2025_14838_MOESM1_ESM.tif]
